# Incidence, prevalence, and disease burden of low back pain in China: data from the global burden of disease database

**DOI:** 10.3389/fpubh.2025.1563823

**Published:** 2025-06-16

**Authors:** Xiaodong Zheng, Wenjin Han, Ming Liu, Lijun Liu, Mingtao Ma, Zonglin Ye, Mengqi Ma, Longtan Yu

**Affiliations:** ^1^The First Clinical Medical College of Shandong University of Traditional Chinese Medicine, Jinan, China; ^2^Weifang Hospital of Traditional Chinese Medicine, Weifang, China; ^3^School of Nursing, Xi’an Jiaotong University Health Science Center, Xi’an, China

**Keywords:** low back pain, global burden of disease, disability-adjusted life years, prevalence, incidence

## Abstract

**Objective:**

This study aimed to estimate the incidence, prevalence, and disability-adjusted life years (DALYs) of low back pain (LBP) in China by gender and age based on data from the Global Burden of Disease (GBD) 2021.

**Methods:**

Joinpoint software was used to identify the turning points and significant changes (*p* < 0.05) in the disease burden of LBP from 1990 to 2021, and calculate the average annual percentage change (AAPC) to quantify the overall rate of trend change. The Age Period Queue (APC) model is used to explore the long-term trends and influencing factors of the burden of LBP.

**Results:**

From 1990 to 2021, both incidence and prevalence rates of LBP were higher in women than in men. The incidence of LBP increased with age, and the number of affected individuals began to rise significantly starting in 1995.

**Conclusion:**

In light of the findings regarding the LBP burden in China, more efforts should be directed toward prevention and treatment for women and older adults. Furthermore, there is a need to further promote health awareness throughout the population.

## 1 Introduction

Low back pain (LBP) is a condition characterized by discomfort in the lower back, specifically between the lower ribcage and the crease of the buttocks, with or without sciatica (1). As a prevalent musculoskeletal disorder, LBP significantly impacts quality of life worldwide. It is currently one of the top three leading causes of non-fatal disability (2), which substantially burdens individuals, family economies, and healthcare systems.

China is the world’s most populous country, and the increasing burden of LBP has garnered significant interest from the medical community. However, the national LBP registry remains inadequate, and epidemiological reports are limited. The Global Burden of Diseases, Injuries, and Risk Factors Study (GBD) database includes health statistics from over 200 nations worldwide, providing crucial theoretical guidance for health policy adjustments and formulation in countries around the globe (3–6).

Based on the latest GBD 2021 data, this study aims to analyze the long-term trends of low back pain in China from 1990 to 2021, including the age-standardized incidence rate (ASIR), age-standardized prevalence rate (ASPR), and disability-adjusted life years (DALYs). Among them, ASIR and ASPR represent the population incidence rate and prevalence rate, respectively, after adjusting for age structure (7), while DALYs refer to the total years of life lost due to premature death and years lived with disability due to epidemic or health issues in the population (8). The findings aim to provide targeted guidance for public health policies in China to mitigate the impact of LBP on individuals and society in the coming decades.

## 2 Methods

### 2.1 Overview

Data for this study were sourced from the GBD database for the year 2021. The GBD database is a collaborative initiative launched by the World Health Organization (WHO), the World Bank, the Bill & Melinda Gates Foundation, various academic institutions, research organizations, and government-led programs. Since its inception in 1990, the GBD database has been continuously maintained and updated, drawing from over 90,000 sources, including research studies and government publications. GBD 2021 uses a standardized global approach to estimate key disease indicators, such as ASIR, ASPR, mortality, and DALYs.

This study focused on ASIR, ASPR, and DALYs associated with LBP, which were systematically analyzed using R programming to provide insights into global and regional trends and impacts of LBP. The analysis did not include mortality rates since LBP does not directly lead to mortality. All figures and tables in this study were generated based on a database. For specific information, please visit https://vizhub.healthdata.org/gbd-compare/.

### 2.2 Joinpoint regression analysis

Joinpoint regression is a statistical method that involves a set of linear models to analyze the trends in disease burden caused by LBP over time. This model is particularly useful for identifying changes in trends and detecting turning points, or “joinpoints,” where the direction of the trend shifts. The methodology behind Joinpoint regression uses least squares estimation to model the changes in disease rates over time. This approach is beneficial because it overcomes the potential subjectivity of typical linear trend analyses. The Joinpoint model identifies points at which the trend undergoes a significant shift by calculating the sum of squared residuals (the squared differences between estimated and observed values).

We used the Joinpoint software (version 4.9.1.0; National Cancer Institute, Rockville, Maryland, USA) to develop the regression model for this analysis. Joinpoint software is widely adopted for analyzing trends in public health data, particularly in epidemiology. Furthermore, we computed the average annual percent change (AAPC), which quantifies the average rate of change in the disease burden throughout the entire study period. This measure is critical for understanding overall trends in disease rates. We compared the AAPC to 0 using hypothesis testing to assess the statistical significance of the trends. A *p*-value of less than 0.05 was considered statistically significant, indicating that the observed trend was unlikely to have occurred by chance. In summary, Joinpoint regression was employed to examine the temporal trends in the disease burden of low back pain, providing insights into whether the burden has been increasing, decreasing, or remaining stable over time, and identifying significant shifts in these trends.

### 2.3 Age-period-cohort analysis

The age-period-cohort (APC) model is commonly used to analyze time trends of incidence or mortality rates based on the Poisson distribution. Due to the linear relationship between age, period, and cohort, Carstensen explained the APC model analysis method through Lexis diagrams (9, 10), which is applied in this study. The research used data from the GBD database, querying the incidence, mortality rates, and annual population estimates from 1990 to 2021 for each 5-year age group. Since the GBD groups individuals aged 0–4 and 95+, this study defined the age groups as 5–9, 10–14, and 95 and older. The study calculated the total number of cases, cumulative incidence, and mortality rates for each age group and period (e.g., 1992–1996, 1997–2001, etc.), and the APC model fitting was performed using R (version 4.2.3). The net drift representatives of the overall linear trend in disease rates after adjusting for period and cohort effects. This parameter is a crucial output of the APC model, reflecting the changing trend of a certain disease during a specific time period.

## 3 Results

### 3.1 Descriptive analysis

In 2021, a total of 100,093,746 people (95% CI: 87,128,173 to 113,014,316) in China were affected by LBP, with 43,374,995 new cases (95% CI: 37,494,376 to 49,159,184) reported. The ASIR, ASPR, and DALYs for LBP were 5,342.1 cases (95% CI: 4,660.41 to 5,976.28) per 100,000, 2,342.46 cases (95% CI: 2,058.05, 2,639.32) per 100,000, and 603.03 DALYs (95% CI: 427.63, 810.16) per 100,000, respectively (Table 1). Table 1 lists cases for all-age cases along with age-standardized rates (ASR) of incidence, prevalence, and DALYs for both males and females. It is evident that the disease burden for females is higher than for males.

**Table 1:** Incidence, prevalence, and DALYs of LBP in China in 2021.

Measure	All-age cases			ASR (per 100,000)		
	Total	Male	Female	Total	Male	Female
Incidence	43,374,995 (37,494,376, 49,159,184)	17,158,052 (14,905,295, 19,511,511)	26,216,943 (22,616,551, 29,717,547)	2342.46 (2058.05, 2639.32)	1901.62 (1673.22, 2155.4)	2779.16 (2436.19,3121.16)
Prevalence	100,093,746 (87,128,173, 113,014,316)	39,148,537 (33,850,361, 44,326,367)	60,945,208 (52,941,431, 68,505,911)	5342.1 (4660.41, 5976.28)	4282.3 (3759.62, 4838.55)	6381.38 (5567.92,7153.22)
DALYs	11,297,805 (7,931,468, 15,328,056)	4,474,521 (3,156,562, 6,067,442)	6,823,284 (4,775,816, 9,218,929)	603.03 (427.63, 810.16)	488.36 (346.52, 657.58)	716.15 (506.26,959.84)

**Figure 1:**
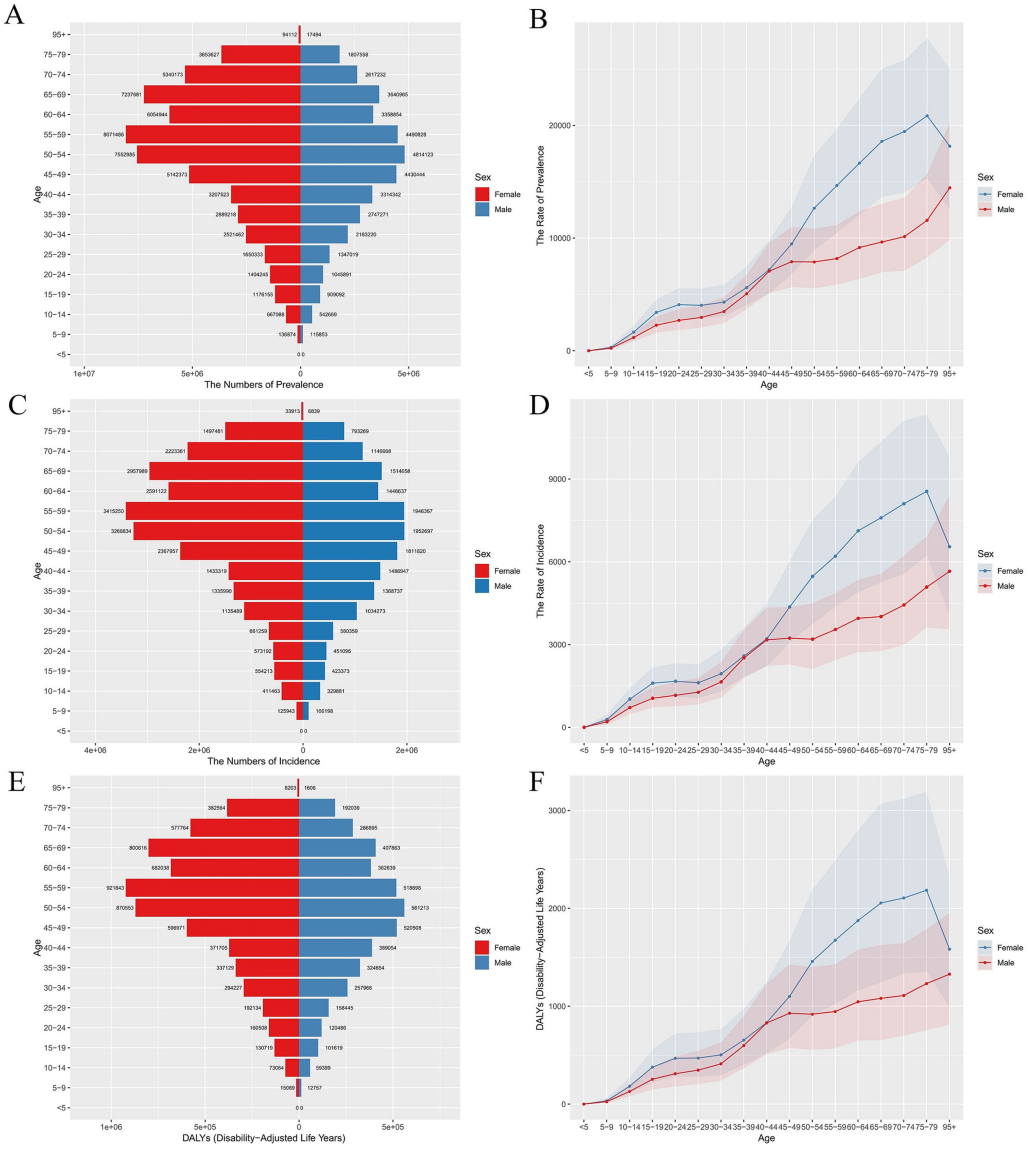
ASPR, ASIR, and DALYs for LBP in China in 2021. **(A)** Biaxial graph of ASPR **(B)**, Line chart of ASPR **(C)**, Biaxial graph of ASIR **(D)**, Line chart of ASIR **(E)**, Biaxial graph of DALYs, and **(F)** Line chart of DALYs.

Figure 1 illustrates the prevalence and incidence rates of LBP in different age groups in 2021 (A, C) and the ASR (B, D). LBP is more common in the population aged 5 years and older, with a sharp increase observed between the ages of 45 and 59. Among women, the impact is more pronounced between the ages of 45 and 69, whereas for men, the highest impact occurs between the ages of 45 and 59. The incidence rate indicates that the onset of LBP is more common after age 45, with the peak incidence occurring between the ages of 45 and 79. The results show that women have higher prevalence and incidence rates across all age groups compared to men. As shown in E and F of Figure 1, due to the higher prevalence and incidence rates in women, the DALYs are also significantly higher for women than for men.

**Figure 2:**
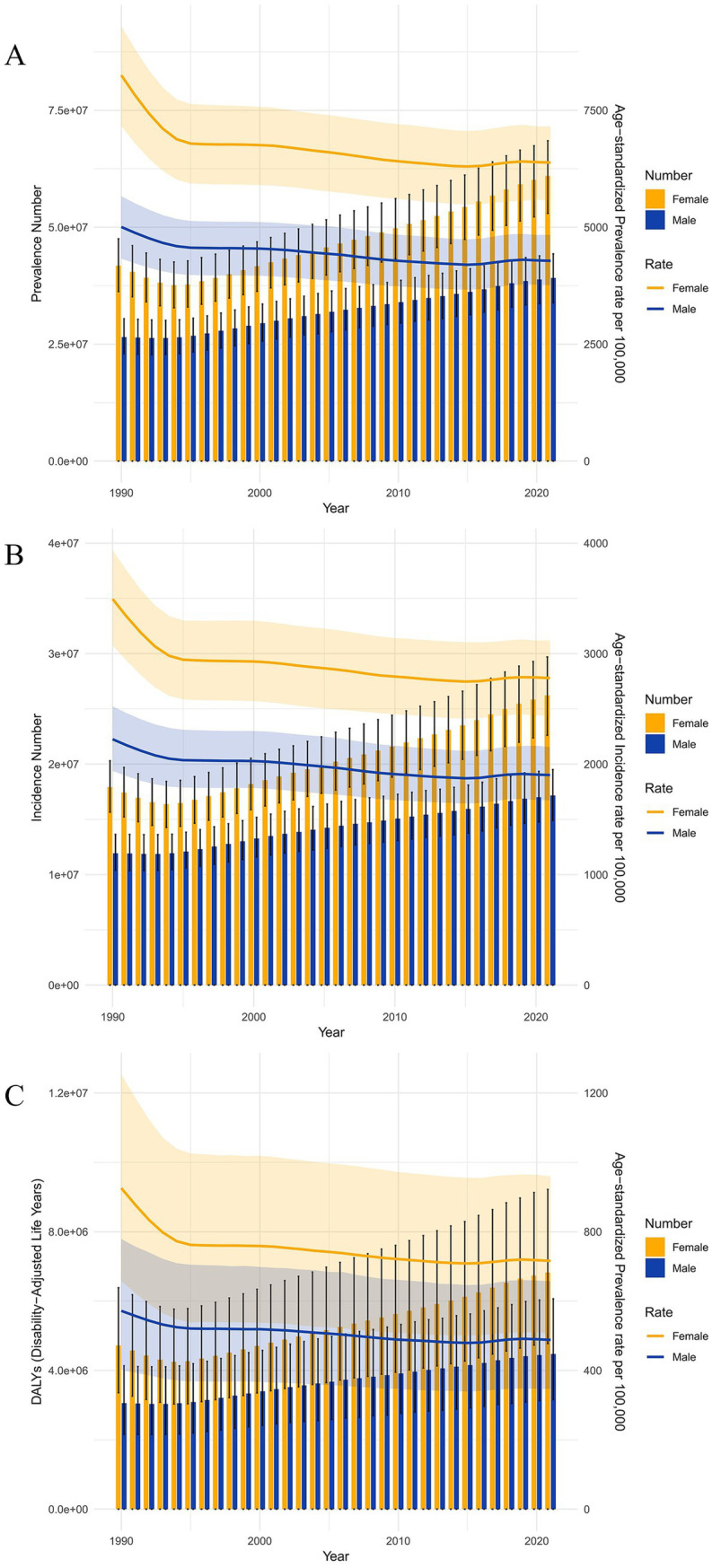
Trends in number and rate of prevalence, incidence, and DALYs of LBP from 1990 to 2021. **(A)** Number and rate of prevalence. **(B)** Number and rate of incidence. **(C)** Number and rate of DALYs.

Figure 2 describes the trends in gender-specific all-age cases, ASIR, and ASPR of LBP in China from 1990 to 2021. Starting in 1990, the prevalence and incidence rates for both men and women exhibited a downward trend, especially with the most significant decrease between 1990 and 1995. From 1996 onwards, the rates tend to stabilize. Since 1990, both the total number and the proportion of DALYs for men and women have remained relatively stable.

### 3.2 Joinpoint regression analysis

**Figure 3:**
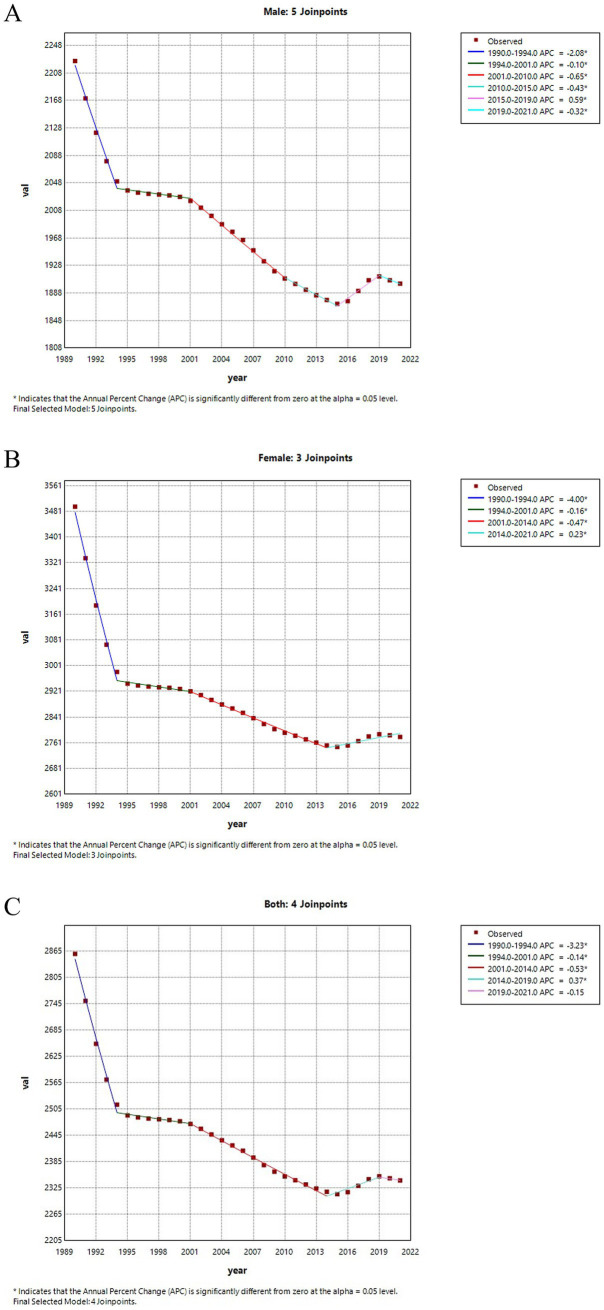
Joinpoint regression analysis of ASIR for LBP in China from 1990 to 2021. **(A)** The ASIR for males. **(B)** The ASIR for females. **(C)** The ASIR for all genders.

**Figure 4:**
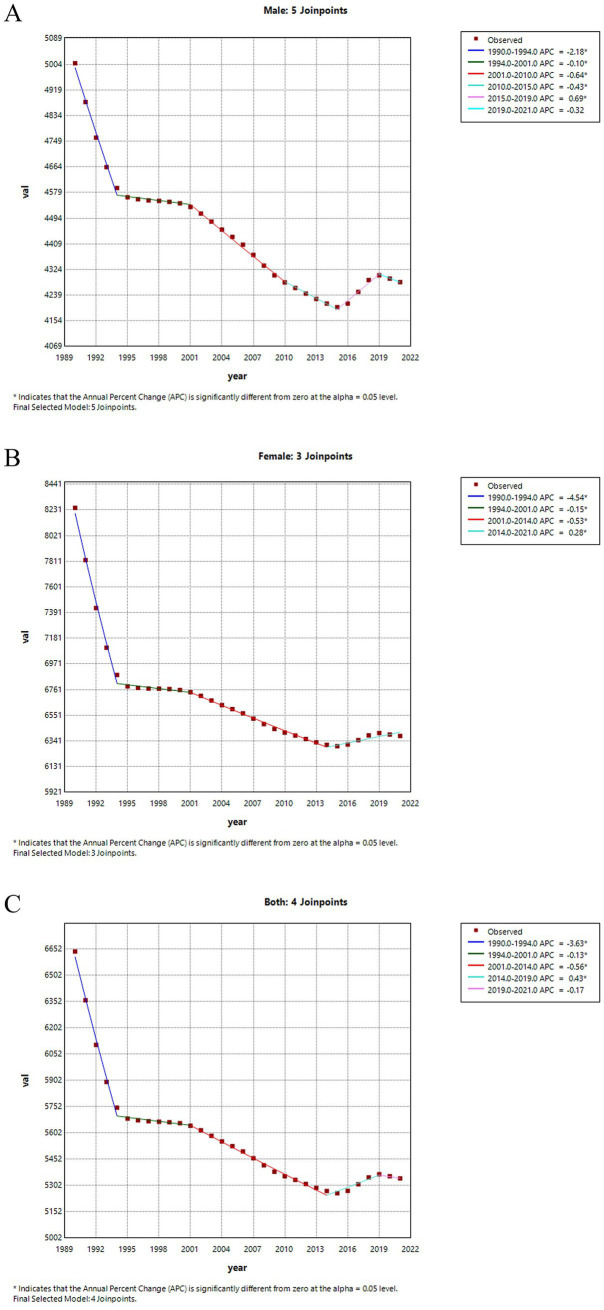
Joinpoint regression analysis of ASPR for LBP in China from 1990 to 2021. **(A)** The ASPR for males. **(B)** The ASPR for all genders. **(C)** The ASPR rate for females.

Figure 3 illustrates the joinpoint regression analysis of ASIR for LBP in China from 1990 to 2021. A significant decreasing trend in incidence rates is observed for both males and females, particularly between 1990 and 1994. The rate for males decreased by-2.0815 (95% CI: −2.1812, −1.9816), whereas for females, it decreased by-3.9998 (95% CI: −4.1762, −3.8232). Although there was a brief upward trend from 2016 to 2019, the rates began to decline again from 2019 onwards. Figure 4 presents the joinpoint regression analysis of the ASPR of LBP in China from 1990 to 2021. It shows that the ASPR in China also exhibited a significant downward trend from 1990 to 2021, roughly the same as the incidence rate.

Table 2 shows the AAPC in the incidence and prevalence rates of LBP over the past 30 years. From 1990 to 2021, the ASIR for LBP in China decreased by-0.6271 (95% CI: −0.6712, −0.5831), while the prevalence rate declined by-0.6817 (95% CI: −0.7278, −0.6355). Notably, the AAPC for incidence and prevalence rates was more favorable for males than females (Table 2).

**Table 2:** Joinpoint regression analysis: trends in ASIR and ASPR for males and females in China from 1990 to 2021.

ASIR				ASPR		
Gender	Period	APC (95% CI)	AAPC (95%CI)	Period	APC (95% CI)	AAPC (95% CI)
Both	1990–1994	−3.2289 (−3.3711, −3.0864)	−0.6271 (−0.6712, −0.5831)	1990–1994	−3.6263 (−3.7774, −3.4750)	−0.6817 (−0.7278, −0.6355)
	1994–2001	−0.1436 (−0.2212, −0.0658)		1994–2001	−0.1336 (−0.2146, −0.0525)	
	2001–2014	−0.5309 (−0.5582, −0.5036)		2001–2014	−0.5619 (−0.5906, −0.5332)	
	2014–2019	0.3716 (0.2246, 0.5188)		2014–2019	0.4345 (0.2806, 0.5887)	
	2019–2021	−0.1513 (−0.6147, 0.3143)		2019–2021	−0.1683 (−0.6512, 0.3168)	
Female	1990–1994	−3.9998 (−4.1762, −3.8232)	−0.7081 (−0.7454, −0.6707)	1990–1994	−4.5421 (−4.7468, −4.3369)	−0.7912 (−0.8339, −0.7485)
	1994–2001	−0.1647 (−0.2634, −0.0660)		1994–2001	−0.1537 (−0.2659, −0.0414)	
	2001–2014	−0.4743 (−0.5092, −0.4393)		2001–2014	−0.5285 (−0.5675, −0.4895)	
	2014–2021	0.2338 (0.1547, 0.3129)		2014–2021	0.2768 (0.1877, 0.3659)	
Male	1990–1994	−2.0815 (−2.1812, −1.9816)	−0.4976 (−0.5329, −0.4622)	1990–1994	−2.1850 (−2.2902, −2.0796)	−0.4944 (−0.5316, −0.4572)
	1994–2001	−0.1011 (−0.1541, −0.0480)		1994–2001	−0.0968 (−0.1523, −0.0413)	
	2001–2010	−0.6505 (−0.6849, −0.6161)		2001–2010	−0.6423 (−0.6785, −0.6061)	
	2010–2015	−0.4306 (−0.5297, −0.3314)		2010–2015	−0.4307 (−0.5361, −0.3252)	
	2015–2019	0.5858 (0.4296, 0.7422)		2015–2019	0.6882 (0.5211, 0.8556)	
	2019–2021	−0.3226 (−0.6361, −0.0081)		2019–2021	−0.3166 (−0.6401, 0.0079)	

**Figure 5:**
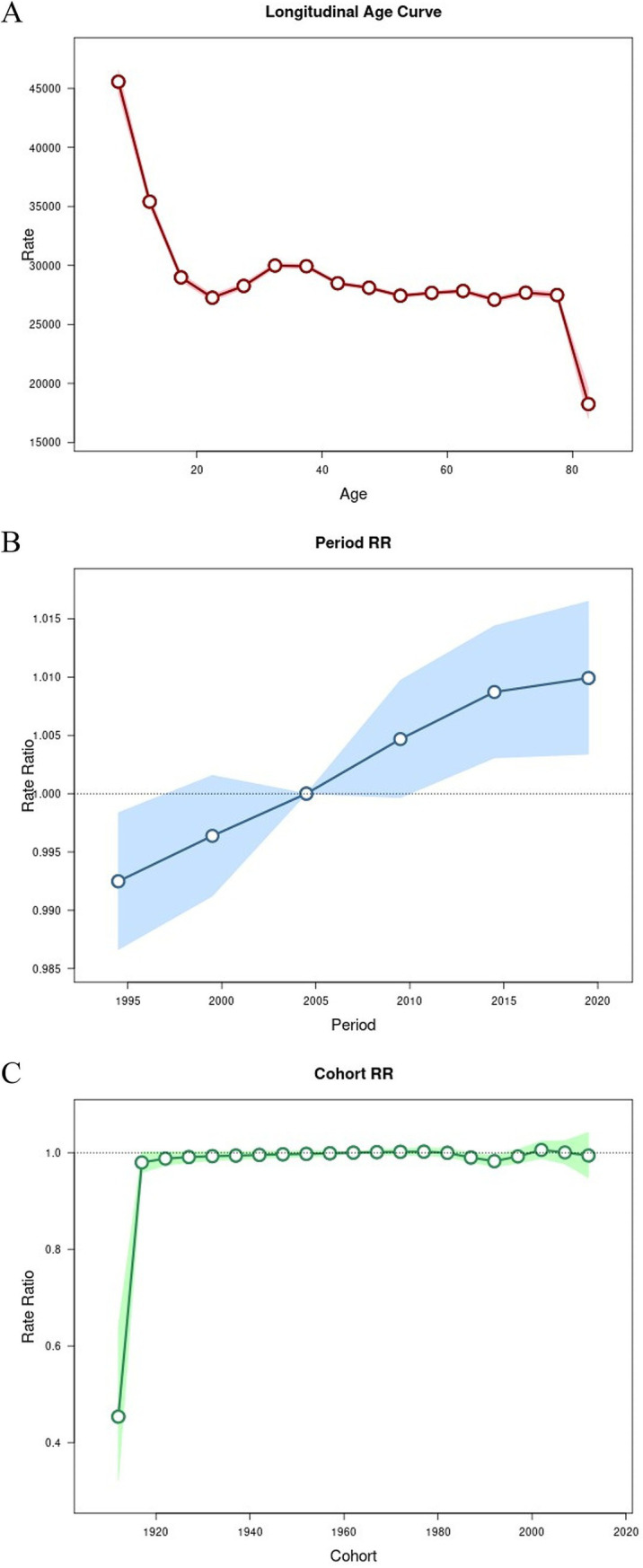
APC analysis of LBP incidence in China. **(A)** Age effect on incidence. **(B)** Period effect on incidence. **(C)** Cohort effect on incidence.

As shown in Table 3, the net drift value for LBP in China is 0.073 (95% CI: 0.039, 0.108). Figure 5 indicates that although there is a brief upward trend in the 20–40 age group, the overall trend remains downward. Over time, the incidence of LBP in China has increased annually. However, the rate of increase has slowed down since 2005. The cohort effect showed that after rising to around 1 in 1920, the value remained relatively stable, showing little fluctuation and staying close to 1.

**Table 3:** Statistical parameters for the APC model data of LBP.

Type	Net drift (% per years; 95% CI)	*p*-value		
		All local drifts = Net drift	All cohort deviations = 0	All period deviations = 0
Incidence	0.073 (0.039, 0.108)	<0.01	<0.01	<0.01

### 3.3 Decomposition analysis

**Figure 6:**
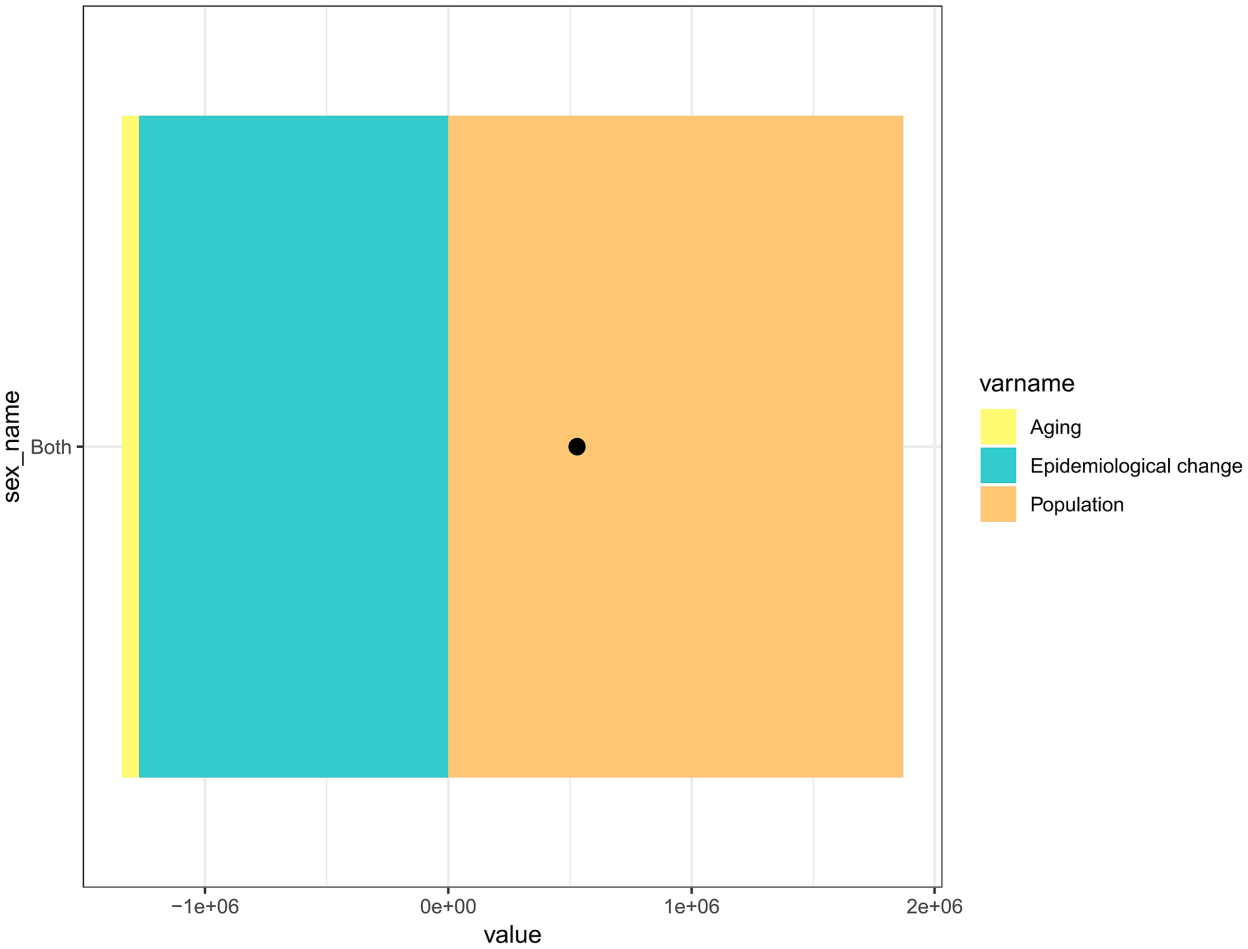
Decomposition analysis of LBP for all genders in China.

Figure 6 presents the analysis of the causes of LBP incidence in China from 1990 to 2021, categorized by age, epidemiological changes, and population size. The figure shows that the increase in population, particularly the growth of the aging population, has greatly contributed to the incidence of LBP. Conversely, the figure represents age and epidemiological changes as negative values, indicating that these two factors do not play a major role in developing LBP incidence and may even have a negative effect.

## 4 Discussion

This study is based on the GBD 2021 database, from which relevant data were extracted to systematically analyze the incidence, prevalence, and DALYs of LBP in China using various analytical methods. According to the 2021 data, the prevalence and incidence of LBP were found to be significantly higher in women than in men, a finding consistent with previous research (11–13). However, to date, we know very little about the causes of this phenomenon (11). Currently, the majority of people believe that women are more affected by skeletal diseases compared to men and are more prone to experiencing pain (14). Gender differences are not just simply distinctions between men and women, but they encompass psychological, biological, and socio-cultural factors (15). From a psychological perspective, women are also more willing to express their experience of pain than men (16, 17). Furthermore, the frequent fluctuations in the menstrual cycle and the hormonal mechanisms that differ from those in men can, to some extent, help explain the differences between men and women (18). Moreover, the hormonal changes that occur during menopause, as well as the increased weight burden during pregnancy, can affect both the skeletal system and the nervous system, leading to the development of LBP (19).

By analyzing the prevalence, incidence, and DALYs across different age groups, we found that the gender difference in LBP is most pronounced in the 50–59 age group, with the number of women significantly exceeding that of men. This phenomenon may be related to the physiological onset of the perimenopausal period in women, which leads to an increase in the prevalence and incidence of LBP among them (20). Previous studies have shown that the median age of menopause onset in women is 51.3 years (21). During menopause, women experience a significant decrease in estrogen levels. According to relevant studies, the reduction in estrogen is closely linked to intervertebral disc degeneration, which is a major cause of LBP (22, 23).

By analyzing data from all genders in China for the year 2021, the number of people experiencing LBP and the incidence rate among both women and men generally showed an increasing trend with age. The following factors may explain this trend. On one hand, intervertebral discs undergo continuous degeneration with age, and disc degeneration is considered a major cause of LBP (24–26). On the other hand, aging is closely related to pain (27). Aging leads to the degeneration of the entire musculoskeletal system, and pain can occur throughout the body. The aging process accelerates the deterioration of muscles and bones, which triggers additional pain in other areas (28).

Although the incidence of LBP among all genders in China has shown a declining trend from 1990 to 2021, the number of patients has continuously increased since 1996, following a brief decline from 1990 to 1995. This rise can be attributed to several factors. First, around 1995, the Chinese government relaxed restrictions on population mobility (29). This policy allowed many people from impoverished areas to migrate to relatively wealthier urban regions for heavy physical labor, significantly increasing the likelihood of developing LBP (30). Another important factor is the development of socioeconomic and technological transformation, particularly with the arrival of the Internet, which has significantly changed people’s work patterns. An increasing number of people are now required to work in front of computers, leading to longer periods of sitting and more frequent instances of sedentary behavior (31, 32). This is also a significant reason for the continuous increase in the number of people suffering from LBP (33).

A decomposition analysis of the incidence data in China from 1990 to 2021 revealed that demographic factors significantly contribute to the increase in LBP cases. This is primarily due to population aging, as China faces the most severe aging problem in the world (34). The continuously increasing aging population has also led to a steady rise in the number of people suffering from LBP in China. While the number of people suffering from LBP in China continues to rise, the ongoing development of the social economy and a growing awareness of health among the public imply that more young people are choosing to engage in light physical labor (35, 36). This has also led to a decline in the incidence of LBP.

The existing prevention strategies have proven effective in preventing and reducing the incidence of LBP. For pregnant women, evidence indicates that moderate physical exercise is well accepted in this population and demonstrates long-term efficacy in LBP prevention (37). For workers engaged in prolonged high-intensity physical labor, the use of ergonomic tools or assistive devices during occupational activities has shown beneficial effects in lowering LBP occurrence (38). Regarding therapeutic interventions across all age groups, exercise therapy is recommended as a first-line treatment for chronic LBP, demonstrating superior efficacy compared to isolated rest or pharmacological management alone (39). In addition to the above-mentioned preventive measures, the national side should integrate LBP into the national primary health care system, and at the social level, it should strengthen the education and training regarding women’s health knowledge, as well as promote and strengthen the application of ergonomic devices in the workplace.

This study has several limitations. First, it uses the GBD database, with the earliest data from 1990 and the latest from 2021 due to the global pandemic. Therefore, this study is limited by a single data source and the time range of the GBD database. Second, this study only discussed the prevalence, incidence rate, and DALYs of LBP in China, lacking provincial analysis and an examination of treatment and etiology of LBP (such as occupational risk factors). Finally, this study may have biases; for example, the database may include a wide range of specific LBP cases but has limitations for “non-specific” LBP, and there may be misclassification bias in ICD codes in the source data.

In conclusion, our results provide a detailed overview of the changes in LBP in China over the past two decades and offer guidance for future disease prevention. The focus of future efforts can be directed toward women and older adults to reduce their disease burden.

## Data Availability

All data included in this study are available upon request by contacting the corresponding author.
